# Extracting higher-conductivity designs for solid polymer electrolytes by quantum-inspired annealing†

**DOI:** 10.1039/d3ra01982a

**Published:** 2023-05-15

**Authors:** Kan Hatakeyama-Sato, Yasuei Uchima, Takahiro Kashikawa, Koichi Kimura, Kenichi Oyaizu

**Affiliations:** a Department of Applied Chemistry, Waseda University Tokyo 169-8555 Japan oyaizu@waseda.jp; b Fujitsu Ltd Kanagawa 211-8588 Japan

## Abstract

Data-driven optimal structure exploration has become a hot topic in materials for energy-related devices. However, this method is still challenging due to the insufficient prediction accuracy of material properties and large exploration space for candidate structures. We propose a data trend analysis system for materials using quantum-inspired annealing. Structure–property relationships are learned by a hybrid decision tree and quadratic regression algorithm. Then, ideal solutions to maximize the property are explored by a Fujitsu Digital Annealer, which is unique hardware that can quickly extract promising solutions from the ample search space. The system's validity is investigated with an experimental study examining solid polymer electrolytes as potential components for solid-state lithium-ion batteries. A new trithiocarbonate polymer electrolyte offers a conductivity of 10^−6^ S cm^−1^ at room temperature, even though it is in a glassy state. Molecular design through data science will enable accelerated exploration of functional materials for energy-related devices.

## Abstract 2

A quantum-inspired annealing system with a hybrid algorithm accelerates functional material discovery, shown by high-conductivity polymer electrolytes.

## Graphical abstract

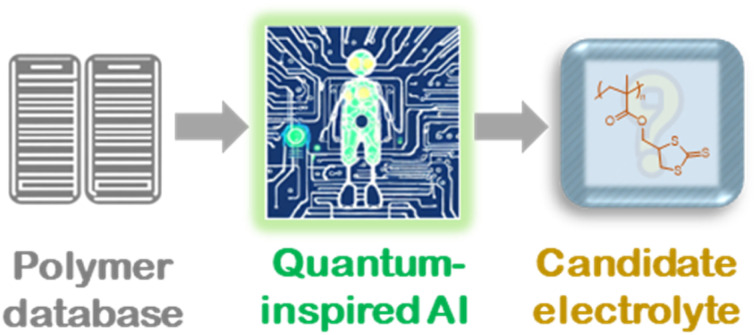

## 1 Introduction

Data-driven materials exploration has become a key technology in materials science.^1–3^ Unlike human researchers, computers can obtain an overview of huge material structure databases and extract universal trends. New photoluminescent compounds,^4^ solar cell components,^5^ battery electrolytes,^6,7^ and energy-related materials have been extracted through data science.^3,8–13^*In silico* material screening is expected to accelerate research cycles and also is compatible with automated experiments.^14–17^

**Fig. 1:**
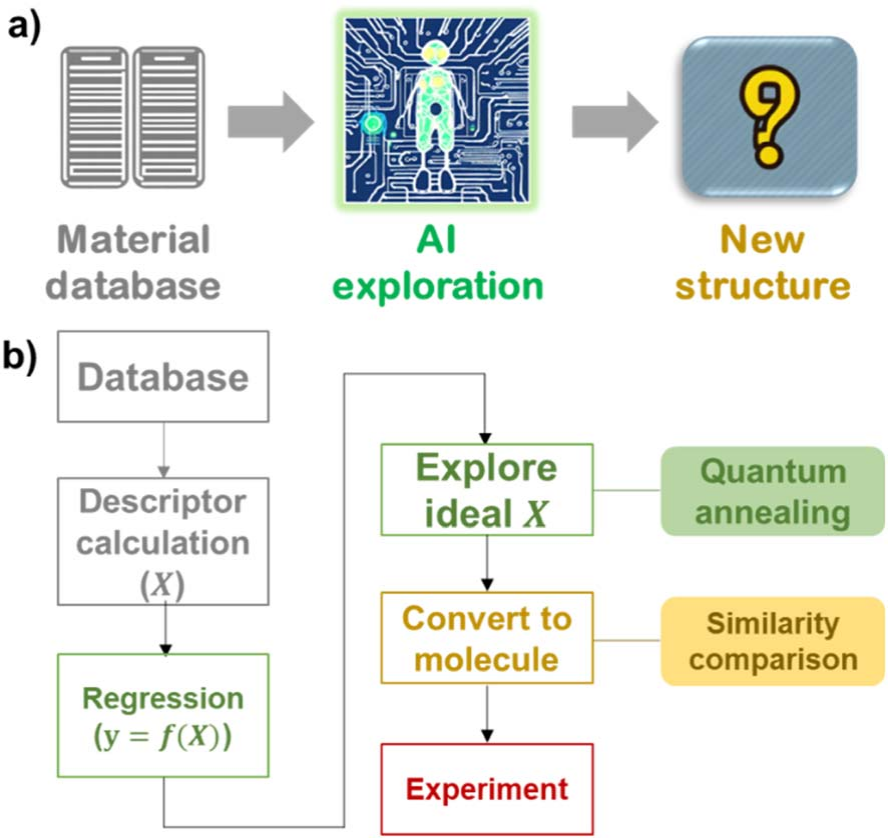
(a) Scheme of material exploration by artificial intelligence. (b) Protocol to extract candidate molecular structures by quantum-inspired annealing in this work.

Artificial intelligence-based material screening consists of three main steps: (1) database construction, (2) structure exploration, and (3) experimental investigation (Fig. 1a).^3,18,19^ The most challenging part of informatics is efficient structure exploration. Promising candidates must be extracted from the limited number of existing material datasets. An accurate prediction model must be constructed, and the exploration system must find reasonable structures from a huge search space of materials. For example, there are potentially more than 10^60^ types of structures for organic compounds, but regular computers cannot explore each candidate in a reasonable time.^20–23^

Exploring an ideal material structure, *X*, with a given parameter, *y*, is called an inverse problem.^23,24^ It has become easy to construct a normal prediction model *y* = *f*(*X*) with conventional machine learning models implemented in, for example, scikit-learn libraries.^25^ In contrast, the construction of the inverse function, *f*^−1^, is still difficult due to scientific and technological challenges. First, the relationship between *X* and *y* is not bijective; specific material structure *X* determines property *y*, but there can be many materials that may have the same *y*.^23,26^ Therefore, most studies use forward function *f* and examine different *X* to search better structures with optimal *y*.^1,2,18^ The second problem is that even defining prediction model *f* is difficult, especially in experimental projects where trainable databases are smaller.^3^ Every model contains prediction errors due to insufficient trainable data and the approximation of prediction algorithms. Third, extensive forward prediction with different structures *X* takes a long time due to the astronomical number of candidates, and thus the exploration space must be carefully reduced.^20,21^

Several algorithms have been proposed to solve inverse problems for materials. Deep reinforcement learning searches for suitable structures that satisfy specific target properties, and these algorithms are often developed for organic molecules, especially drugs.^27^ The exploration space is often reduced by specifying the pre-trained molecular structures in the deep learning models.^21,27^ Another deep learning algorithm, chemical autoencoder, reversibly converts chemical structures to continuous vectors,^12,28,29^ and structure exploration becomes an optimization problem in the multidimensional vector space.^12^ Recently, quantum annealing and the hardware that it has inspired have been used to explore large search spaces efficiently by superparallel computing.^20,21,30,31^

Despite major algorithmic and hardware developments, most technologies still have gaps for experimental molecular screening projects. The difficulties arise not only from insufficient prediction accuracy of a target parameter, but also from molecule filtering by practical criteria.^21^ Molecules must satisfy good synthetic accessibility, stability, safety, cost, and other parameters.^21^ Individual modules have been developed to evaluate the parameters, especially for drugs, but effective integration has been difficult due to problems with prediction accuracy and computational cost.^32,33^ Considering the current technical level of molecular screening for materials science, the suggestions may be used as indicators rather than exact solutions.

Here, we demonstrate a molecule exploration system that provides a near-ideal design for molecules to improve a specific parameter, *y* (Fig. 1b). A forward prediction model of *y* = *f*(*X*) was constructed from a structure–property relationship database of organic functional molecules. Then, the ideal vector, *X*, was searched for by a Fujitsu Digital Annealer, which is quantum-inspired annealing hardware.^34,35^ The hardware overcomes the intrinsic difficulties of real quantum machines, such as the limited dimensions of the search space and the restriction options during computation, but its computation time is reasonable (about 1 to 10 s). Comparing ideal *X* with existing molecules revealed essential features for improving the performance of materials. The exploration system was used to design a new solid-state polymer electrolyte for lithium-ion batteries. An emerging trithiocarbonate design for the electrolyte yielded a room-temperature conductivity of over 10^−6^ S cm^−1^ and a high glass transition temperature of 100–125 °C. The quantum-inspired annealing algorithm may help researchers design other functional materials for energy-related devices and accelerate research and development.

## 2 Experimental section

### 2.1 Molecule exploration by quantum-inspired annealing

#### 2.1.1 Database preparation and data conversion

We used our open database and program for lithium-ion conducting polymers and electrolytes, which were developed in our previous work.^3,6^ The database and code for processing electrolytes into molecular descriptors are available in an open repository (https://github.com/KanHatakeyama/ion_predictor). The database contains tens of thousands of electrolyte data at various temperatures. An electrolyte consists of single or multiple chemical structures, and several hundred composite electrolytes are maintained in the database. In this work, electrolyte data measured at room temperature (20–30 °C) were extracted, reducing the records to *ca.* 200. It is essential to consider the large changes in ionic conductivity within the range of 20–30 °C. In this study, we did not apply data processing due to the small differences in measurement temperatures across the literature. Instead, we used the reported values directly. Although excluding the temperature factors may affect the reliability of the predictive model, we believe that it does not affect the overall results substantially because we used the model primarily for examining the general structure–property relationships rather than precise predictions.

The chemical structures, recorded as simplified molecular input line entry system (SMILES) strings, were converted into about 200-dimensional numerical descriptors by the RDKit module.^36^ During data processing, up to six compounds were extracted in order of weight per electrolyte, resulting in an approximately 200 × 6 = 1200-dimensional vector. The compound information expressed as the vector was treated as *X* and the logarithmic ionic conductivity was set as *y*. For machine learning, the dimensionality of *X* was reduced to about 200 by dropping columns with the same values in all different records. The variables were standardized as *z*-scores.

#### 2.1.2 Building regression model *y* = *f*(*X*)

The conductivity prediction model, *y* = *f*(*X*), was built by different approaches. The baselines were a random forest regression (RFR; implemented by scikit-learn 1.0.2), the Ridge linear model, and a quadratic regression model. Default hyperparameters were chosen unless noted otherwise. RFR is a powerful model that provides good predictive performance with versatile databases. Ridge is a standard linear model with L2 norm. A quadratic model operating with L2 norm was chosen because of the nature of quantum annealing. The main hyperparameters of alpha for L2 norm and tree depth for RFR were optimized *via* the 10-fold cross validation with the training dataset (Table S1†).^37,38^

Quantum annealing hardware solves quadratic unconstrained binary optimization (QUBO) problems. Solutions that give the minimum in eqn (1) should be explored.^30,34,35^1
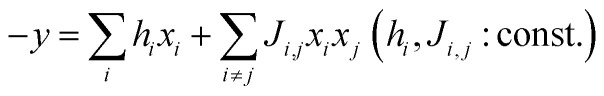


If we build a quadratic regression model according to eqn (1), the quantum annealer finds a solution, *X*_ideal_, that maximizes *y* predicted in the equation. However, *X*_ideal_ is not easily found with normal nonlinear models, such as RFR.

Quadratic terms *J*_*i*,*j*_ in the quadratic model were generated by randomly selecting 500 interactions between *x*_*i*_ and *x*_*j*_. The Ridge algorithm was selected to determine the coefficients. Although the factorization machine algorithm can prepare adequate quadratic interaction terms with low computational costs,^31^ it cannot be used in this work because continuous variables are used for *x*_*i*_, whereas the algorithm only uses binary data as the inputs.

Quantum annealers also only use binary inputs. Thus, a continuous variable, *x*_*i*_, was expressed as 10 bit data (unary method, eqn (2)). Constant *c*_bin,*k*_ was set so that *x*_*i*_ could take between the maximum and minimum values of the training data.2
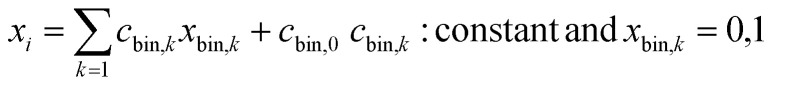


Normal quantum annealers have difficulty in solving these problems because the number of assigned variables is large, and the applicable interactions, (*i*,*j*), are limited due to hardware problems.^39^ In contrast, the third-generation digital annealer can solve up to 100 000 bits with arbitrary interactions,^34^ which is suitable for the current work.

**Fig. 2:**
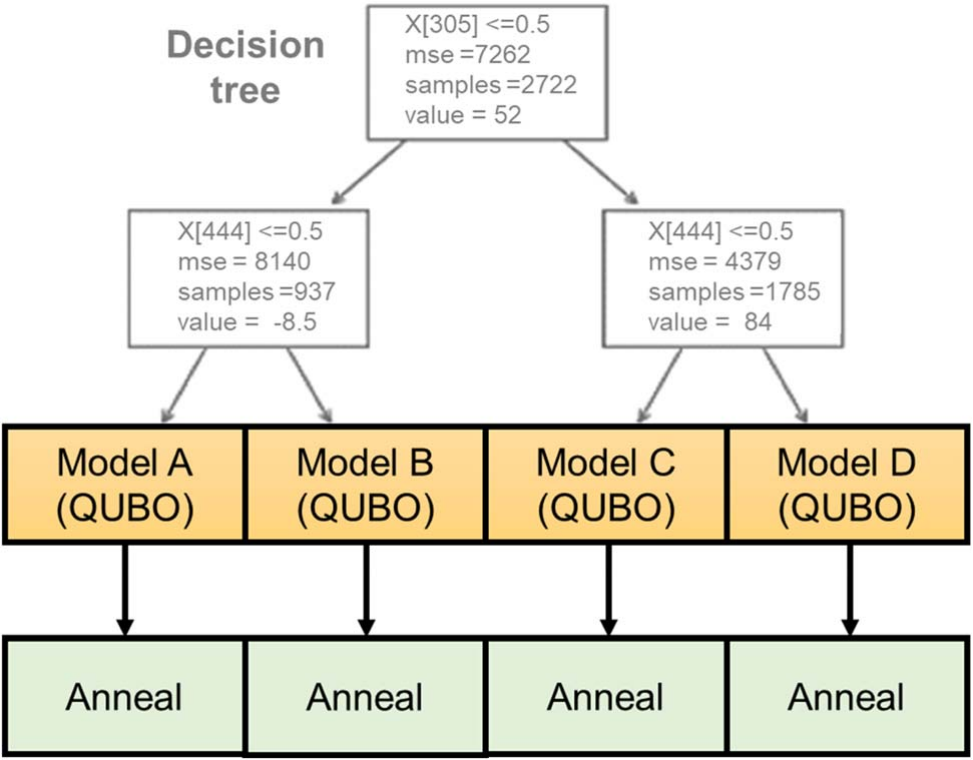
Hybrid regression model of decision tree and quadratic regressions.

Here, a new regression model was developed that is compatible with the annealer (Fig. 2). The algorithm consists of a decision tree and several quadratic regressors. The tree categorizes the input into different groups according to the magnitude of *y*. The depth of the tree was set to two, and the training data were categorized into three groups automatically. Then, individual quadratic models were generated for the different groups. Compared with ordinary quadratic regression, the hybrid model can handle data that is more complex because of the tree. A previously reported tree-linear hybrid model (linear tree)^40^ was not used due to the excessive training time for high-dimensional data. The third-generation Fujitsu Digital Annealer natively handles the constraint conditions generated by the decision tree, whereas normal quantum annealers only input pure QUBO data.

#### 2.1.3 Extraction of chemical structures close to ideal solutions

Solutions *X*_ideal_ giving large *y* were explored from the QUBO data generated by the hybrid model. Then, the binary data were converted into continuous variables by eqn (2). By calculating cosine similarities between *X*_ideal_ and vectors in the existing datasets, chemical structures close to the ideal solutions were extracted. The extracted structures were expected to have essential features for achieving high conductivities. From the analysis data, the trithiocarbonate design was extracted as a candidate structure for solid polymer electrolytes. The screening process is described in the Results and discussion subsection “Chemical structure exploration using quantum-inspired annealing”.

### 2.2 Examination of a trithiocarbonate-based polymer electrolyte

#### 2.2.1 Polymer synthesis

All chemical reagents were used as received unless otherwise noted. Poly(trithiocarbonate-substituted methacrylate) (PTCMA) was designed as a matrix for a lithium-ion conducting polymer electrolyte. The polymer was synthesized according to a previous report with modifications.^41^ The monomer (0.73 g, 3.10 mmol), *N*-methyl-2-pyrrolidone (3.8 mL, Tokyo Chemical Industry Co.), and 2,2′-azobis(2-methylpropionitrile) (15.6 mg, 0.10 mmol, Tokyo Chemical Industry Co.; recrystallized from methanol before use) were added to a 10 mL round flask and freeze degassed. The mixture was stirred at 60 °C for 24 h to allow radical polymerization to proceed. After the reaction was complete, the product was purified by precipitation in methanol and dried in a vacuum overnight to afford PTCMA as a yellow powder (*M*_n_ = 3.2 × 10^4^, *M*_w_ = 5.9 × 10^4^, *M*_w_/*M*_n_ = 1.8, 82% yield). Differential scanning calorimetry (DSC) estimated the glass transition temperature to be around 117 °C, which was comparable to a previously reported value of 121 °C.^41^

#### 2.2.2 Electrolyte examination

Samples were prepared in an argon-filled glove box. PTCMA (30 ± 0.5 mg) and lithium bis(trifluoromethanesulfonyl)imide (LiTFSI; Kanto Chemical Co.) were added to *N*,*N*-dimethylformamide (DMF; 1 mL) at different molar ratios and stirred overnight. The salt concentrations were 5, 11, 25, 33, and 50 mol%. Concentrations higher than 50 mol% were not accessible because the resulting film became brittle. The solution was dropped onto a stainless-steel plate, air-dried, and vacuum-heated overnight (<1 mbar, 100 °C). After capping with another stainless-steel plate, the sample was annealed at 150 °C for 10 min. The sample was placed in a conventional solid-state device. Its impedance was evaluated with an impedance analyser (ZAHNER CIMPS, Zahner Co. or 1260, Solartron) under open-circuit potential at frequencies from 10^6^ to 1 Hz. DSC was performed with a conventional calorimeter (Q200, TA Instruments) at a scan rate of 10 °C min^−1^.

## 3 Results and discussion

### 3.1 Regression model construction

**Fig. 3:**
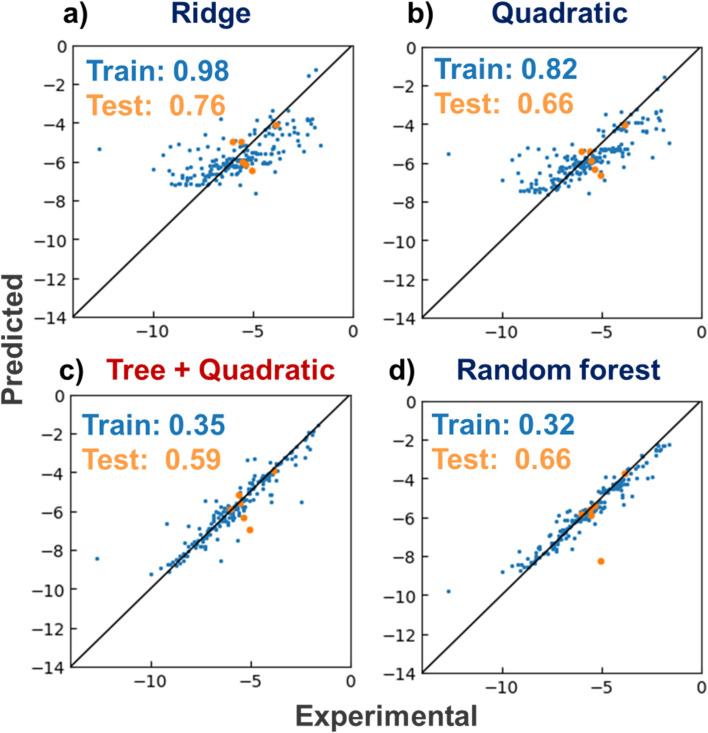
Prediction of logarithmic ionic conductivities (S cm^−1^) of lithium-ion conducting electrolytes. *X*-and *Y*-axes show experimental and predicted values, respectively. (a) Ridge, (b) quadratic, (c) tree plus quadratic, and (d) random forest regressors. MAEs for training and testing datasets are shown in the plots. Data reported prior to 2018 were used as the training dataset, and data reported in early 2019 were used as the test dataset.

The linear Ridge, RFR, quadratic, and tree and quadratic hybrid regressor regression models were constructed with a lithium-ion conductive polymer and electrolyte dataset (Fig. 3). The models were trained to predict logarithmic ionic conductivities *y* from their electrolyte structures *X*. As a standard method for quantifying molecular properties, roughly 200-dimensional molecular descriptors were computed by the RDKit module. The training data were electrolytes reported before 2018, and the test data were those reported in early 2019.^6^ The mean absolute errors (MAEs) for the training data were 0.98 (Ridge), 0.82 (quadratic), 0.35 (hybrid), and 0.32 (RFR). The order of the errors was the same as the complexity of the algorithms of RFR > hybrid > quadratic > Ridge. The prediction trend for the test data was similar to that of the training data of 0.76 for Ridge, 0.66 for quadratic, 0.59 for hybrid, and 0.66 for RFR. The new hybrid model afforded the smallest MAE. The model was more straightforward than RFR, but it adequately matched the complexity of the current dataset. Thus, the model offers promising predictive performance for conductivity in virtual molecular screening.

### 3.2 Quantum-inspired annealing

A third-generation Fujitsu Digital Annealer was used to search for optimal solutions to obtain large *y*. The hardware is specialized for solving the QUBO problem expressed in eqn (1). Finding the maximum or the minimum from the equation is challenging because of the complex quadratic interactions between the variables.^21,30,35^ For an *N*-dimensional binary input, 2^*N*^ types of combinations must be examined to find the best solutions. The exponential nature of the problem leads to an astronomically large search space (*e.g.*, 2^100^ ≅ 10^30^).

Quantum annealers are a new type of computer based on quantum physics and can solve QUBO problems efficiently.^34,35,39^ Their superparallel nature can outperform classical computers, although the acceptable dimensions of data and interactions are currently limited.^39^ Thus, quantum-inspired classical hardware and software have been developed as alternatives to real annealers. These quantum-annealer-mimicking systems, such as the Fujitsu Digital Annealer^34,35^ and simulated bifurcation machines (SBMs),^42^ provide practical solution times of typically 1 to 10^1^ s, and they provide similar solutions to quantum annealers.

A third-generation digital annealer accepts one of the largest binary bit sizes of 100 000,^34^ whereas true (D-Wave: about 5000)^39^ and other (SBM: 10 000)^42^ quantum annealers accept smaller sizes. The higher dimension allows quasi-continuous variables to be used with annealers, even though they accept originally binary data (0 or 1). In the present work, the dimension of the electrolyte structure data *X*was about (200 descriptor dimension) × (maximum of 6 compound components) = 1200. If each continuous variable is expressed with 10 bits by the unary method (eqn (2)), the corresponding bit size would be as large as 12 000. This dimension size is acceptable for the digital annealer, but it can be challenging for D-Wave and SBM.

In the current work, the dimension of the continuous vector *X* was, fortunately, reducible from about 1200 to 200 by dropping the columns in which all the records have the same variables. The hybrid model was still most compatible with the Fujitsu Digital Annealer because the decision tree and quadratic regression model generated QUBO data. The decision tree categorized the training data into three main groups. Then, three QUBO data were generated with different constraints specified by the tree algorithm. With normal annealers, constraints must be carefully embedded in QUBO data by adding large constants, which requires parameter optimization. In contrast, the annealer natively accepts the constraints during solution finding, and we designed the hybrid regression model for this hardware.

### 3.3 Chemical structure exploration using quantum-inspired annealing

Quantum-inspired annealing with the three different QUBO data yielded ideal solutions *X*_ideal_ to allow large conductivities. The sampled solutions were expected to have almost maximum *y* under the specific constraints. The sampled solutions predicted *y* of 6, 57, and −12, corresponding to conductivities of 10^6^, 10^57^, and 10^−12^ S cm^−1^. Because the experimental conductivities were limited to 10^−1^ S cm^−1^, the first two solutions were too large. However, the last solution offered more practical conductivity because the decision tree set appropriate constraints.

**Fig. 4:**
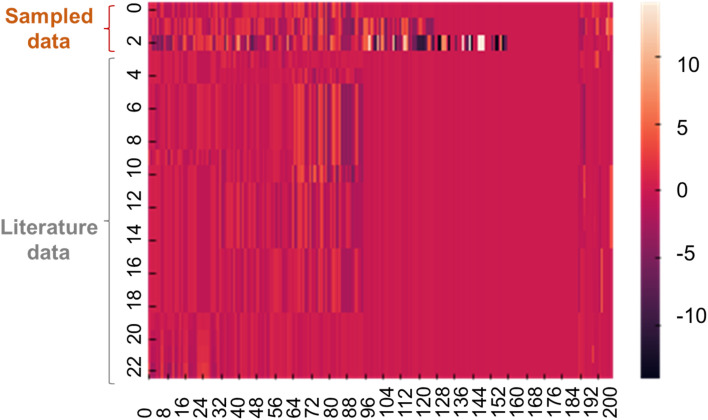
Heatmap for the continuous vector of *X*. The first three lines show data sampled by annealing. The rest are representative records in the training dataset.

A heatmap was created to compare *X*_ideal_ with several datasets from the training data (Fig. 4). Although the first two solutions were infeasible in terms of *y*, they looked like existing data, indicating that the corresponding chemical structures could be reasonable.

According to the sampled solutions, essential chemical structures for higher conductivity were explored. We calculated the cosine similarity between *X*_ideal_ and electrolyte data in the training dataset (Fig. S1†). As the similarity increased, the experimental conductivities tended to increase because *X*_ideal_ contained essential features for larger *y*. The highest similarity was limited to about 70% due to the limited types of chemical structures recorded in the experimental database. Imperfect constraints during quantum sampling were another reason for the gap. We found no significant trend differences in the chemical structures of the three sampled solutions; thus, the essential structural trends should not change significantly, regardless of ionic conductivities (Fig. S2 to S4†).

**Fig. 5:**
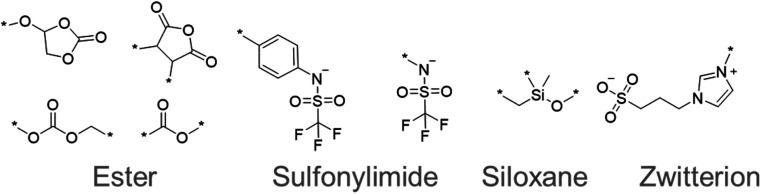
Frequently appearing chemical structures sampled by quantum-inspired annealing.

The chemical structures that appeared frequently are summarized in Fig. 5. The most common species were esters, such as cyclic and linear carbonates.^43,44^ The esters are popular electrolyte components for lithium-ion batteries because their polar moieties solvate ions. Sulfonylimide anions were also extracted many times because their delocalized negative charge allows greater dissociation of ions and higher ionic conductivity. Flexible siloxane structures also appeared as candidates. They are often used with solid-state polymer electrolytes because they exhibit lower glass transition temperatures, and thus allow more active ion movement.^43,44^ Furthermore, zwitterionic salts have been proposed as additives to electrolytes and their highly polarized molecular structure has attracted attention for superionic conduction.^45^ These results demonstrated that our system can extract important and reasonable molecular structure trends for higher performance. Such comprehensive trend analysis has been done mainly by humans but is also now accessible by computers.

Suggesting *de novo* molecular structures is a challenging topic in informatics. Just comparing solutions with conventional molecular data does not yield new molecular designs. However, new molecular designs can be obtained from the sampled solutions by using deep reinforcement learning, although it requires additional computation and parameter tuning.^21^ This work used a heuristic approach, involving manually modifying molecules from the extracted candidates.

**Fig. 6:**
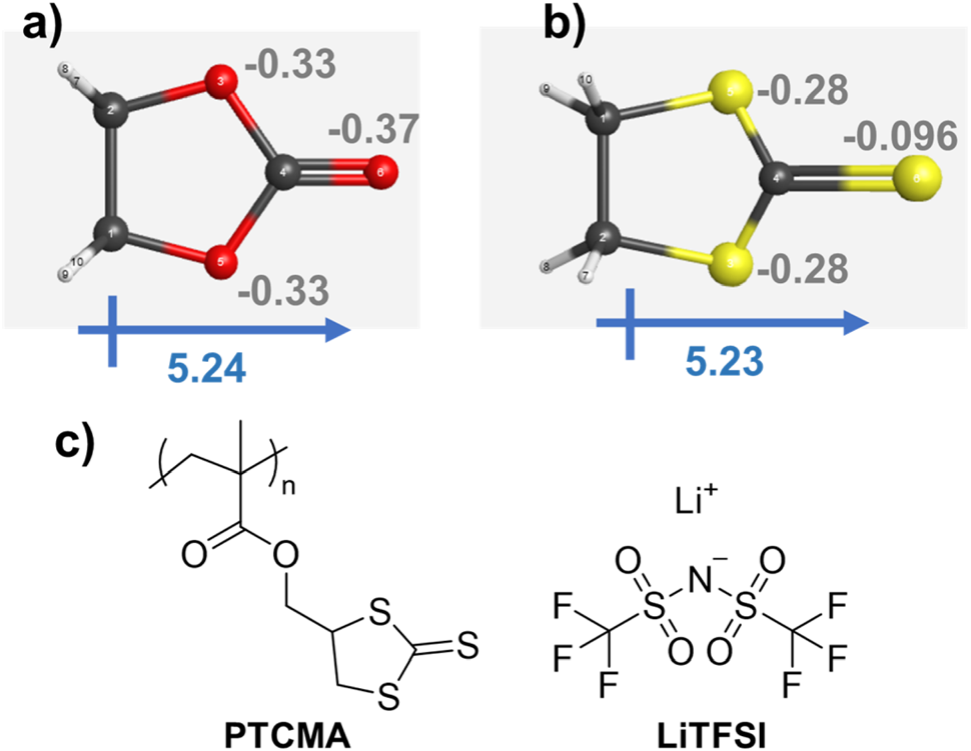
Three-dimensional molecular structures of the cyclic (a) carbonate and (b) trithiocarbonate. Gray numbers indicate Mulliken charges. The structures were optimized by DFT calculations using the B3LYP 6-31G(d′,p′) level in a vacuum. (c) Chemical structures of PTCMA and LiTFSI.

A cyclic carbonate was suggested by quantum-inspired annealing as a reasonable electrolyte design. We decided to replace the oxygen atoms in the ring with sulfurs, yielding trithiocarbonate (Fig. 6). The decision to replace the oxygen atoms in the ester with sulfur atoms was made purely from the perspective of a human chemist. Although our system was helpful in extracting essential information from known data, it was not necessarily beneficial for providing an entirely novel perspective. Therefore, instead of oxygen, we decided to focus on sulfur, which also belongs to group 16 in the periodic table and possesses similar properties but with some differences.

Density functional theory (DFT) calculations by Gaussian16 were performed for the two cycles at the B3LYP 6-31G(d′,p′) level (Fig. 6a and b). The structures were initially optimized by a semiempirical PM6 method, and then by B3LYP 6-31G(d′,p′). The cyclic carbonate and trithiocarbonate molecules yielded large dipole moments of about 5.2, indicating the potential ability to solvate cations.

The Mulliken charge distribution on the molecules was slightly different. Heteroatoms in the two five-membered rings showed a charge of −0.3, whereas the charges on the (thio)ketone differed: the values were −0.37 for oxygen and −0.096 for sulfur. The higher electronegativity of oxygen resulted in a larger negative value on the carbonyl group. Despite the interesting similarities and differences between carbonate and thiocarbonate, thiocarbonate has been little studied as an electrolyte, except as an additive for solid electrolyte interphase formation.^46^

### 3.4 Trithiocarbonate polymer for solid-state electrolytes

**Fig. 7:**
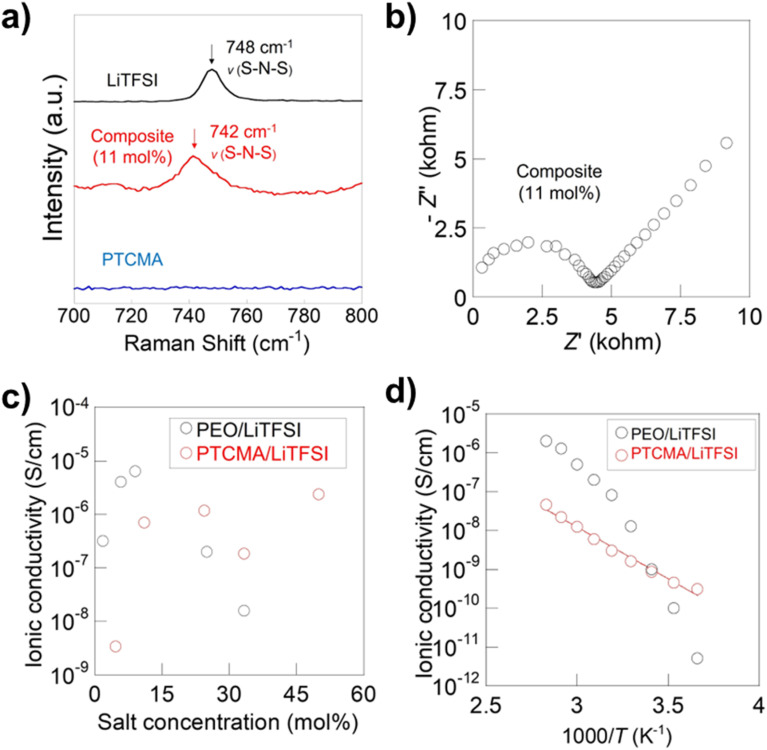
Characterization and conductivity data of the polymer electrolytes. (a) Raman spectra for the electrolyte and pristine components. (b) Nyquist plot for the 11 mol% LiTFSI in PTCMA electrolyte measured at 10^6^ to 10^0^ Hz. See Fig. S5 to S8† for other plot data. (c) Room-temperature conductivity as a function *x*. (d) Arrhenius plot for the 11 mol% electrolyte and a typical PEO electrolyte. Conductivity data for PEO was taken from a previous report.^50^

We synthesized PTCMA as a macromolecular medium for solid-state polymer electrolytes (Fig. 6c). The polymer has a readily synthesizable methacrylate backbone and polar trithiocarbonate side chains. PTCMA has been studied as a high refractive index material,^41^ but not as an electrolyte component. Self-supporting films were prepared by casting a DMF solution of PTCMA and LiTFSI and drying the solvent. Sulfur-containing polymers, such as poly(ethylene sulfide), are poorly soluble in organic solvents due to the high polarity,^47^ but the flexible methacrylate chain contributed to the solubility of PTCMA in DMF. The peak shift of the anion vibration in LiTFSI caused by complexation with the polymer indicated solvation of cations by the polar trithiocarbonate groups (Fig. 7a). The result is consistent with literature reporting the potential of trithiocarbonate to solvate lithium cations.^48,49^ The methacrylate backbones themselves have not been considered as solvating groups for cations.^43^ A composite film of PTCMA and LiTFSI showed typical semicircles for ion conduction in the Nyquist plots (Fig. 7b, and S5 to S8†). We varied the molar concentration of LiTFSI in PTCMA (per unit) (*x* = 5, 11, 25, 33, and 50). The corresponding room-temperature conductivity, *σ*, generally increased from 3 × 10^−9^ to 2 × 10^−6^ S cm^−1^ (Fig. 7c); the higher amount of salt contributed to the higher charge carrier density and conductivity. This response was in sharp contrast to polyethylene oxide (PEO)-based electrolytes.^50^ The traditional composite exhibited maximum conductivity at the LiTFSI concentration of 10 mol% because higher amounts of salts induced greater physical crosslinking.^50^ However, the current sulfur electrolytes did not exhibit the apparent maximum conductivity. The difference may be because sulfur had weaker interactions with lithium atoms than oxygen according to the hard and soft acid and base (HSAB) rule.

The temperature dependence of the conductivity generally followed the Arrhenius equation (Fig. 7d). The activation energy for the 11 mol% electrolyte was 35 kJ mol^−1^, giving a much smaller temperature dependence of conductivity than the PEO composite where ion motion is strongly coupled to the segmental motion of the polymers.^50^

The composite film was mechanically hard even after complexation with lithium salts. A high glass transition temperature above 100 °C was obtained with the 11 mol% salt composite (Fig. S9†). The result was different from a carbonate polymer in which the sulfur atoms in PTCMA were replaced by oxygens, and the plasticizing effect lowered the glass transition temperature to −19 °C.^51^ The weaker interactions of sulfur with lithium than with oxygen according to the HSAB rule may have resulted in the retention of the original mechanical properties. The carbonate polymer exhibited a room-temperature conductivity of 10^−5^ S cm^−1^ with LiTFSI, about 10 times greater than the current electrolyte.^51^ However, the loss in conductivity was slight compared with the drastic difference in glass transition temperatures of >150 °C for trithiocarbonate and −19 °C for carbonate.

Although the conductivity of 10^−6^ S cm^−1^ did not reach that of state-of-the-art solid polymer electrolytes (>10^−4^ S cm^−1^),^43,44^ the characteristics of the PTCMA electrolyte could be promising due to the improved thermal properties. Amorphous electrolytes without crosslinking show high conductivities, but are viscous above the glass transition temperature.^44^ Semicrystalline polymers such as PEO are mechanically tough but the crystalline domains are not highly conductive. PEO also begins to melt at a relatively low temperature of about 70 °C.^44^ Crosslinking is essential to improve thermal and mechanical properties, but interface engineering becomes more challenging due to the decreased miscibility with other materials.^43,44^ Conversely, linear and glassy polymers, including the electrolytes presented here, can have technological advantages because they are thermally stable and easily molded or solution cast. Their decoupled ion movement through hard media is scientifically attractive and even has the potential for superionic conduction.^52,53^ We intend to continue to optimize molecular structures using the molecule exploration system and plan to evaluate better candidates in real battery environments.

## 4 Conclusions

We constructed a molecule exploration system using an artificial intelligence algorithm and a Fujitsu Digital Annealer as solution-finding hardware. The material exploration conditions were automatically set using a supervised machine learning model that learned a database of experimental lithium-ion conducting molecular electrolytes. The model processed the statistical relationship between the electrolyte structures and their conductivities. In contrast to the traditional quadratic regressors compatible with quantum annealing, the current study used a hybrid decision tree and quadratic function algorithm, which resulted in a more complex supervised model. The third-generation digital annealer could natively process constraints generated by the decision tree and quasi-continuous variables. The smooth combination of the hybrid model with the new hardware allowed easy material exploration for improving electrolyte performance.

Inspired by the structures suggested by digital annealing, new solid polymer electrolytes containing cyclic trithiocarbonate moieties were investigated experimentally. Lithium salt composites showed potentially promising ionic conductivities above 10^−6^ S cm^−1^ at room temperature and thermal stabilities above 80 °C. Data-driven molecular screening can objectively extract essential features for higher performance and help design newer structures. More extensive applications with different materials will improve the exploration system and accelerate research to pursue better functional materials for energy-related devices.

## Author contributions

K. H. wrote the code, designed the experiments, and wrote the manuscript. Y. U. performed experiments and analyzed data. T. K. and K. K. contributed to the development of the Fujitsu Digital Annealer. K. O. conceived the project and participated in the discussion.

## Conflicts of interest

There are no conflicts to declare.

## Supplementary Material

RA-013-D3RA01982A-s001
