# Supplementary material for: Extracting higher-conductivity designs for solid polymer electrolytes by quantum-inspired annealing
Source: RSC Adv. 2023 May 15;13(21):14651–9. doi: 10.1039/d3ra01982a (PMC10183718; doi:10.1039/d3ra01982a)
Supplement: RA-013-D3RA01982A-s001 [file RA-013-D3RA01982A-s001.pdf]

Electronic Supplementary Material (ESI) for RSC Advances.
This journal is © The Royal Society of Chemistry 2023

Supplementary Information

Extracting higher-conductivity designs for solid polymer electrolytes by quantum-inspired annealing

Kan Hatakeyama-Sato,* Yasuei Uchima, Takahiro Kashikawa, Koichi Kimura, and Kenichi Oyaizu*

Table S1 Hyperparameter optimization of regression models.

Model	Hyperparameter	MAE
Ridge	$\alpha = 0.1$	2.31
Ridge	$\alpha = 1$	1.87
Ridge	$\alpha = 10$	1.51
Ridge	$\alpha = 100$	1.31
Ridge	$\alpha = 1000$	1.31
Quadratic	$\alpha = 0.1$	2.25
Quadratic	$\alpha = 1$	1.56
Quadratic	$\alpha = 10$	1.16
Quadratic	$\alpha = 100$	1.11
Quadratic	$\alpha = 1000$	1.16
Random forest	max_depth = 3	1.23
Random forest	max_depth = 5	1.21
Random forest	max_depth = 10	1.15
Random forest	max_depth = ∞	1.17

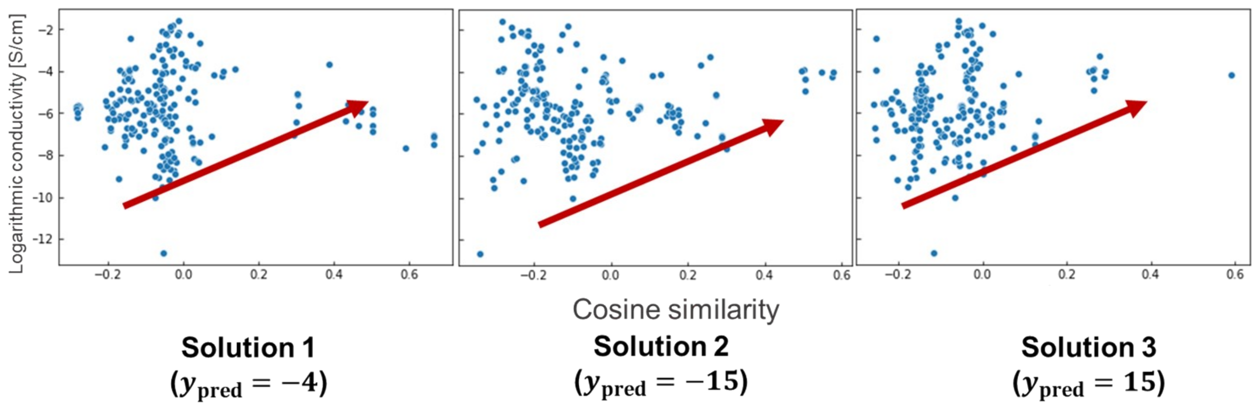

Fig. S1 Relationships between cosine similarity to ideal solutions X_{ideal} and experimental conductivities for the training dataset.

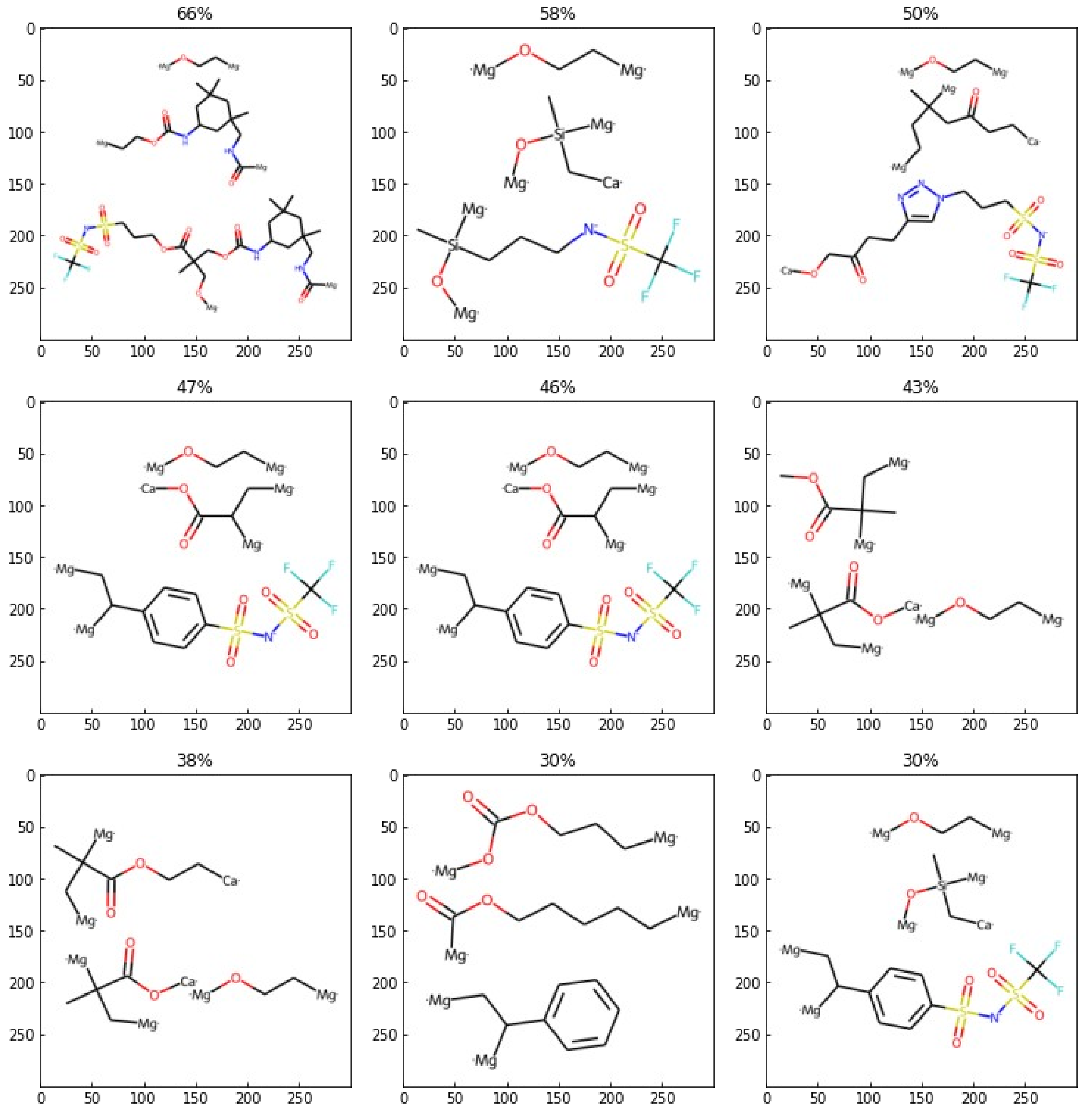

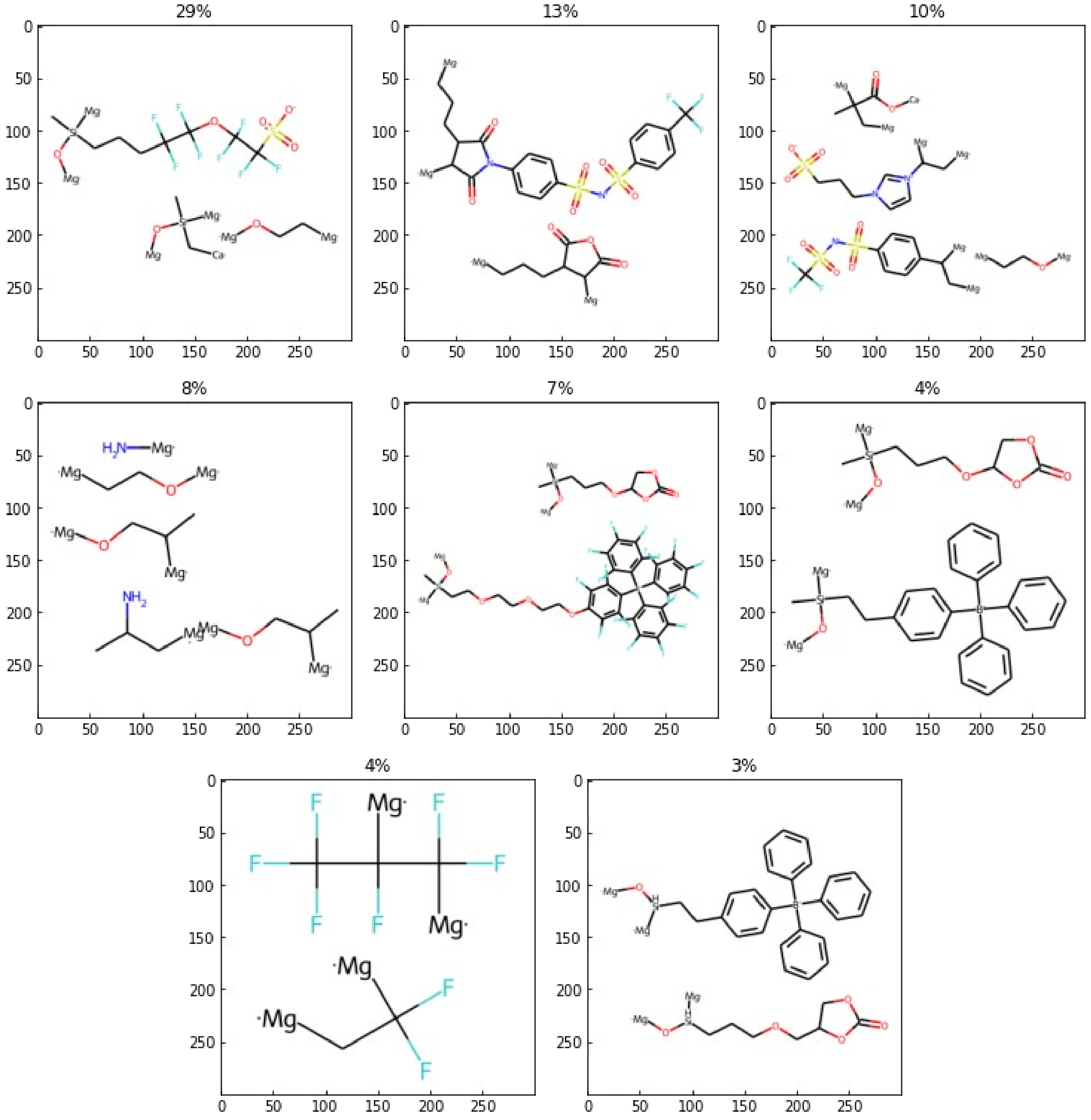

Fig. S2 Electrolyte structures similar to sampled solution 1. Cosine similarities are shown above the structure images. Repeating unit symbols are expressed as Mg or Ca atoms.

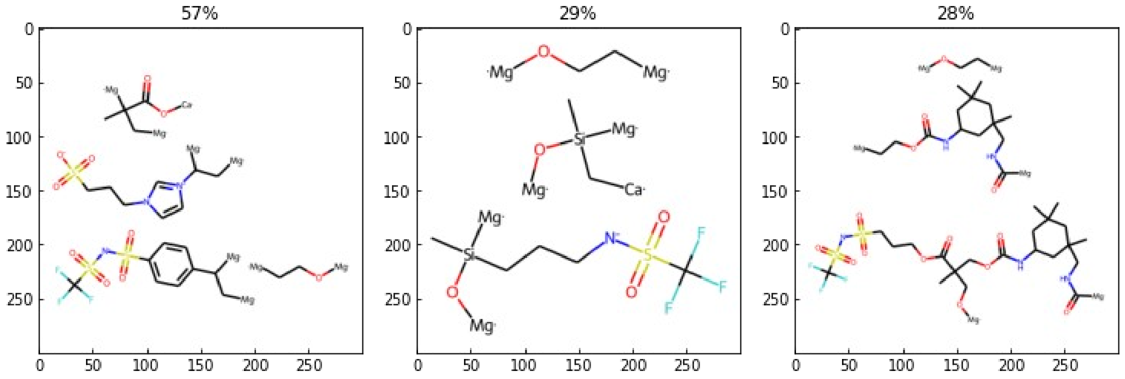

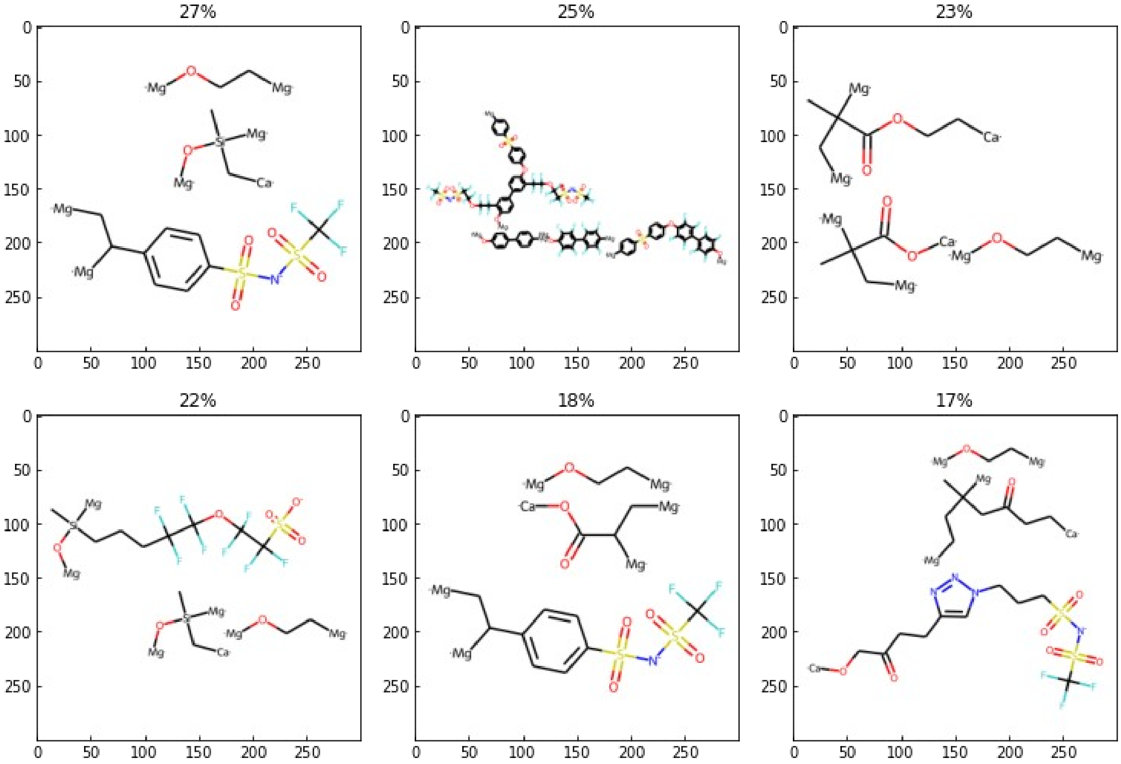

Fig. S3 Electrolyte structures similar to sampled solution 2. Cosine similarities are shown above the structure images. Repeating unit symbols are expressed as Mg or Ca atoms.

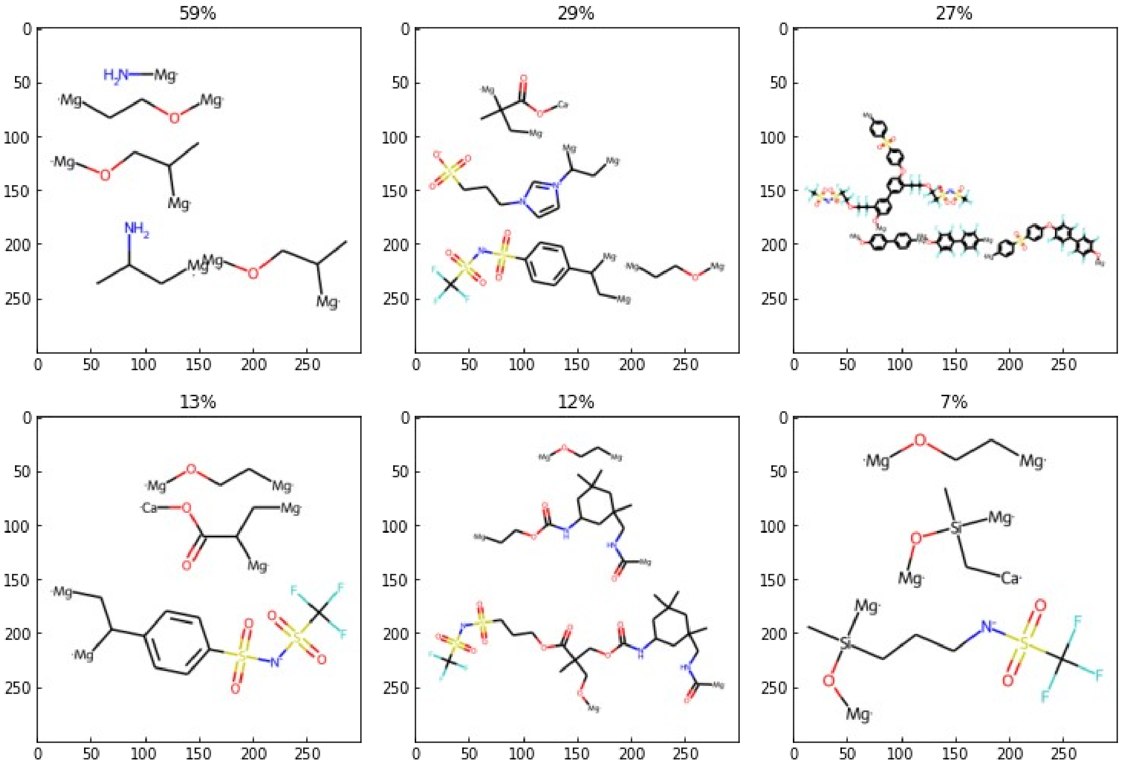

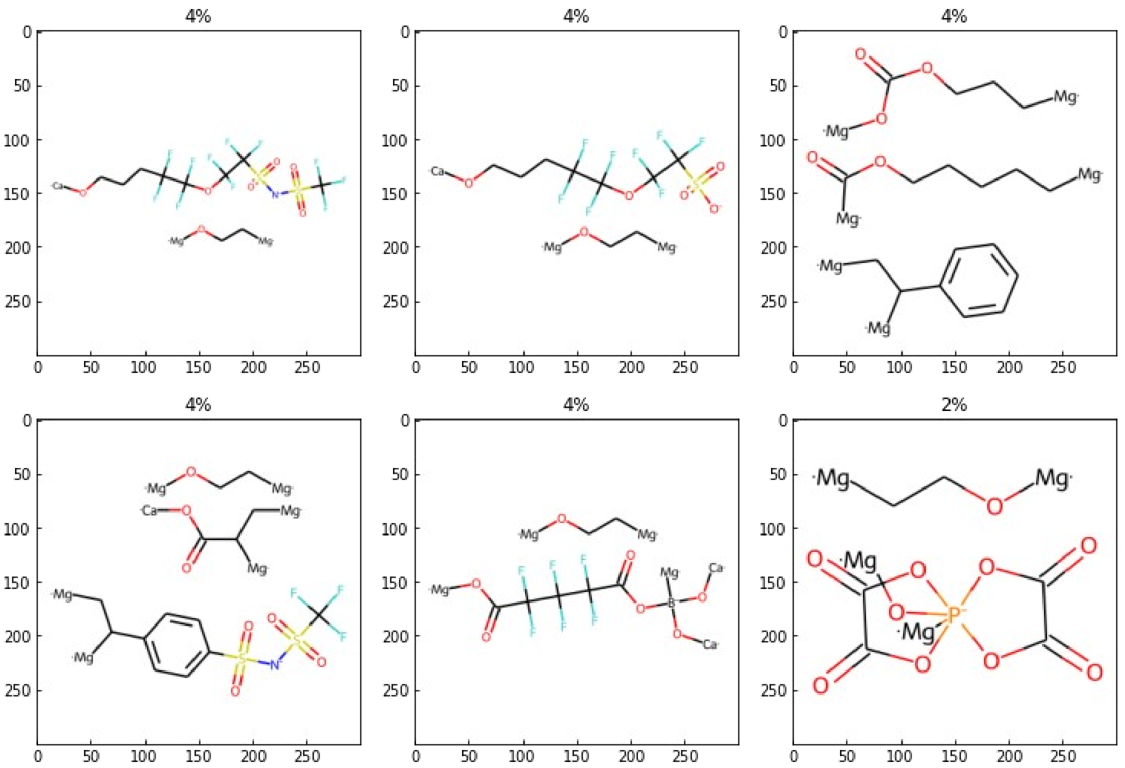

Fig. S4 Electrolyte structures similar to sampled solution 3. Cosine similarities are shown above the structure images.

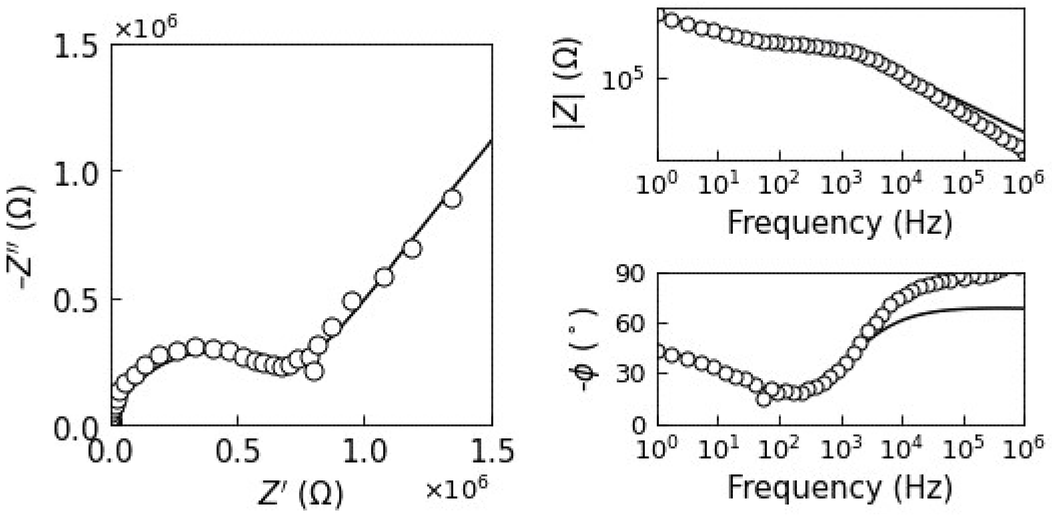

Fig. S5 Nyquist and Bode plots for the 5 mol % polymer composite.

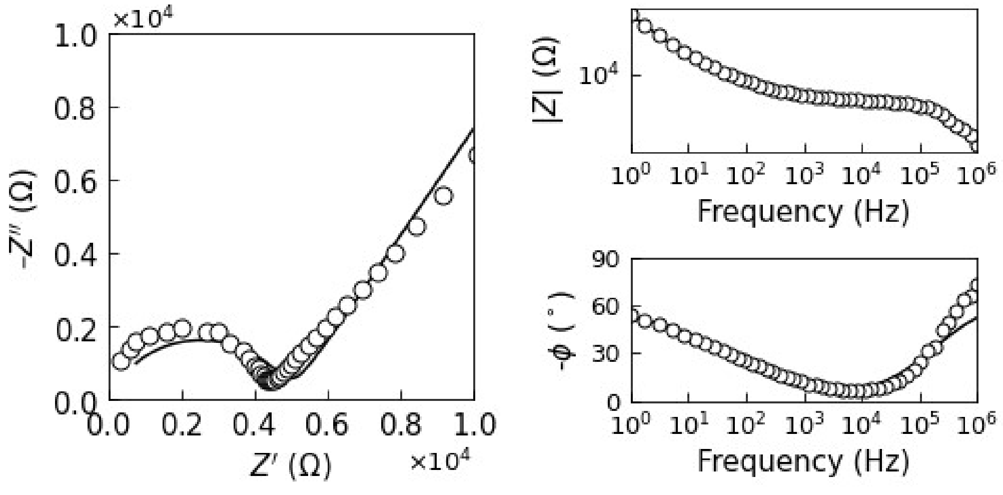

Fig. S6 Nyquist and Bode plots for the 11 mol % polymer composite.

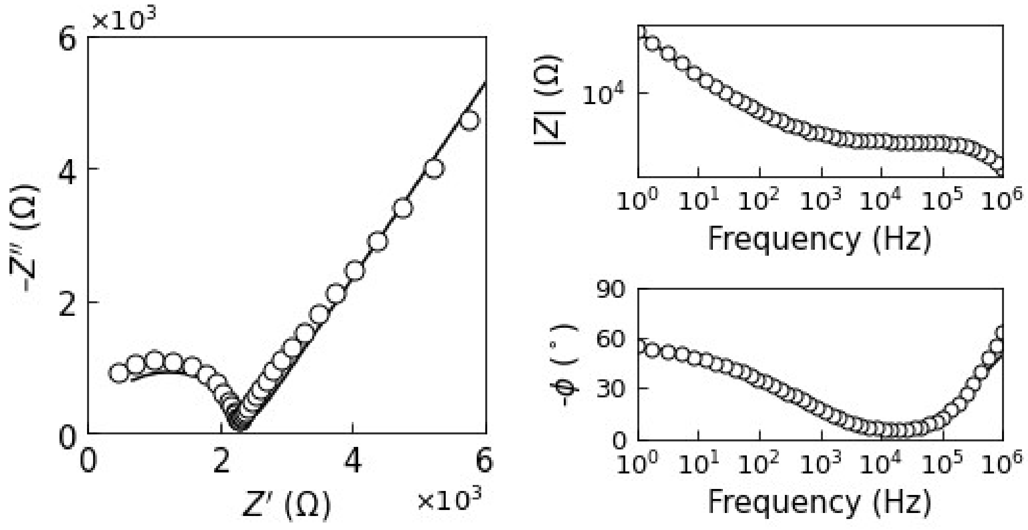

Fig. S7 Nyquist and Bode plots for the 25 mol % polymer composite.

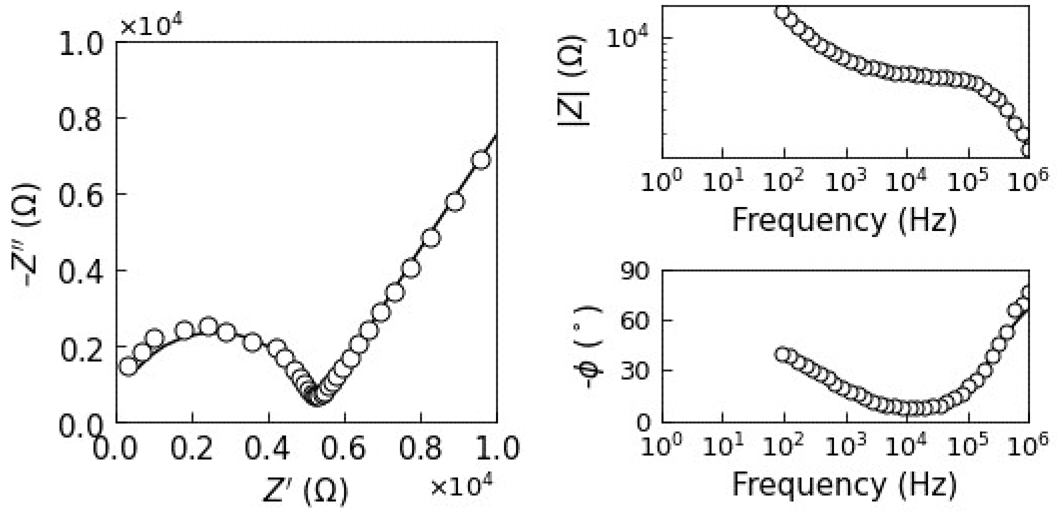

Fig. S8 Nyquist and Bode plots for the 33 mol % polymer composite.

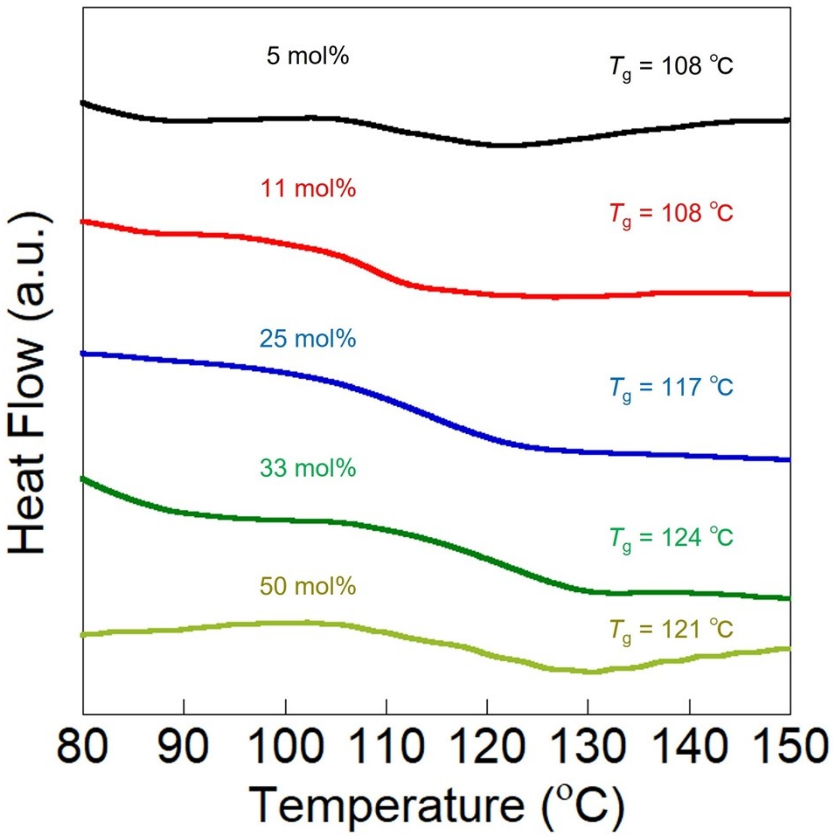

Fig. S9 DSC curve for the different electrolytes.
